# Insights into Novel Choroidal and Retinal Clinical Signs in Neurofibromatosis Type 1

**DOI:** 10.3390/ijms241713481

**Published:** 2023-08-30

**Authors:** Fabiana Mallone, Ludovico Alisi, Luca Lucchino, Valerio Di Martino, Marcella Nebbioso, Marta Armentano, Alessandro Lambiase, Antonietta Moramarco

**Affiliations:** Department of Sense Organs, Sapienza University of Rome, 00161 Rome, Italy; fabiana.mallone@uniroma1.it (F.M.); ludovico.alisi@uniroma1.it (L.A.); luca.lucchino@uniroma1.it (L.L.); valerio.dimartino@uniroma1.it (V.D.M.); marcella.nebbioso@uniroma1.it (M.N.); marta.armentano@uniroma1.it (M.A.); antonietta.moramarco@uniroma1.it (A.M.)

**Keywords:** neurofibromatosis Type 1 (NF1), retinal diseases, genetic mutations, choroidal abnormalities (CAs), NF1 diagnostic criteria, retinal vascular abnormalities (RVAs), optic pathway gliomas (OPGs), SD-OCT (Spectral Domain-Optical coherence tomography), OCT Angiography (OCTA), electrophysiology

## Abstract

Neurofibromatosis type 1 (NF1) is a rare inherited neurocutaneous disorder with a major impact on the skin, nervous system and eyes. The ocular diagnostic hallmarks of this disease include iris Lisch nodules, ocular and eyelid neurofibromas, eyelid café-au-lait spots and optic pathway gliomas (OPGs). In the last years, new manifestations have been identified in the ocular district in NF1 including choroidal abnormalities (CAs), hyperpigmented spots (HSs) and retinal vascular abnormalities (RVAs). Recent advances in multi-modality imaging in ophthalmology have allowed for the improved characterization of these clinical signs. Accordingly, CAs, easily detectable as bright patchy nodules on near-infrared imaging, have recently been added to the revised diagnostic criteria for NF1 due to their high specificity and sensitivity. Furthermore, subclinical alterations of the visual pathways, regardless of the presence of OPGs, have been recently described in NF1, with a primary role of neurofibromin in the myelination process. In this paper, we reviewed the latest progress in the understanding of choroidal and retinal abnormalities in NF1 patients. The clinical significance of the recently revised diagnostic criteria for NF1 is discussed along with new updates in molecular diagnosis. New insights into NF1-related neuro-ophthalmic manifestations are also provided based on electrophysiological and optical coherence tomography (OCT) studies.

## 1. Introduction

Neurofibromatosis Type 1 (NF1; OMIM 613113), formerly called von Recklinghausen disease, is a rare genetic disorder transmitted by autosomal dominant inheritance [1]. Half of NF1 cases are inherited from a parent, while the remaining 50% of patients are the first members of their family to have the condition.

The disease presents with complete penetrance and affects approximately 1 in 3000 individuals worldwide, regardless of gender and ethnicity [2,3,4].

It results from heterozygous pathogenic variants in the NF1 gene located on chromosome 17q11.2 that encode for neurofibromin, a protein acting as a negative regulator of the RAS proto-oncogene. The loss of neurofibromin results in unopposed RAS activity with disruption of normal cell cycle regulation, excessive cell growth and tumorigenesis [5].

The disease pathogenesis involves Schwann cells, neural crest-derived melanocytes, prevertebral ganglia and sympathetic neurons. Nearly all organ systems in the body can be affected by NF1, showing significant clinical heterogeneity and an age-related nature of clinical expression. The main features of NF1 disease include cutaneous, neurological and ocular manifestations and a predisposition to developing multiple benign and malignant neoplasms. NF1 disease may also present as mosaicism, further known as segmental NF1. In these cases, the usual characteristics of disease are preserved but limited to one body region [6].

The eye and adnexa are generally involved in NF1. The diagnostic eye hallmarks of NF1 include optic pathway gliomas (OPGs), iris Lisch nodules, orbital and eyelid neurofibromas and eyelid café-au-lait macules (CALMs) [7].

In recent years, new manifestations have been reported in the ocular system in NF1 including choroidal abnormalities (CAs), hyperpigmented spots (HSs) and retinal vascular abnormalities (RVAs). Also, the recent progress in multimodal imaging in ophthalmology has enabled better characterization of these manifestations. In accordance, CAs have recently been added to the revised diagnostic criteria for NF1 based on high specificity and sensitivity [8,9]. Moreover, there have been recent developments in molecular diagnosis for NF1, adding new insights to our existing knowledge. Additionally, significant progress in electrophysiological and optical coherence tomography (OCT) studies has significantly improved our understanding of NF1-related neuro-ophthalmic manifestations.

In this paper, we review the latest progress in the understanding of choroidal, retinal and neuro-retinal abnormalities in NF1 patients with a focus on the recently revised diagnostic criteria for NF1.

## 2. Choroidal Abnormalities (CAs) in Neurofibromatosis Type 1 (NF1)

Choroidal involvement in NF1 was first reported in histopathologic studies as ovoid bodies in the outer choroid consisting of hyperplastic Schwann cells, neural crest-derived melanocytes and ganglion cells arranged in concentric rings around axons in lamellar pattern [10,11,12]. Based on these histopathological features, CAs were attributed to hamartomatous lesions similar to Lisch nodules of the iris, both sharing the same embryological origin from the neural crest [12].

CAs are completely asymptomatic and not detectable by conventional ophthalmoscopic examination, autofluorescence (FAF) or fluorescein angiography (FA). These anomalies are initially identified as regions exhibiting decreased fluorescence during the initial stages (choroidal stage) of indocyanine green angiography (ICGA). Yasunari et al., using ICGA and infrared monochromatic light with a confocal scanning laser ophthalmoscope (cSLO), identified hypofluorescent patches on indocyanine-green angiograms corresponding to bright, patchy areas on cSLO [13].

**Figure 1:**
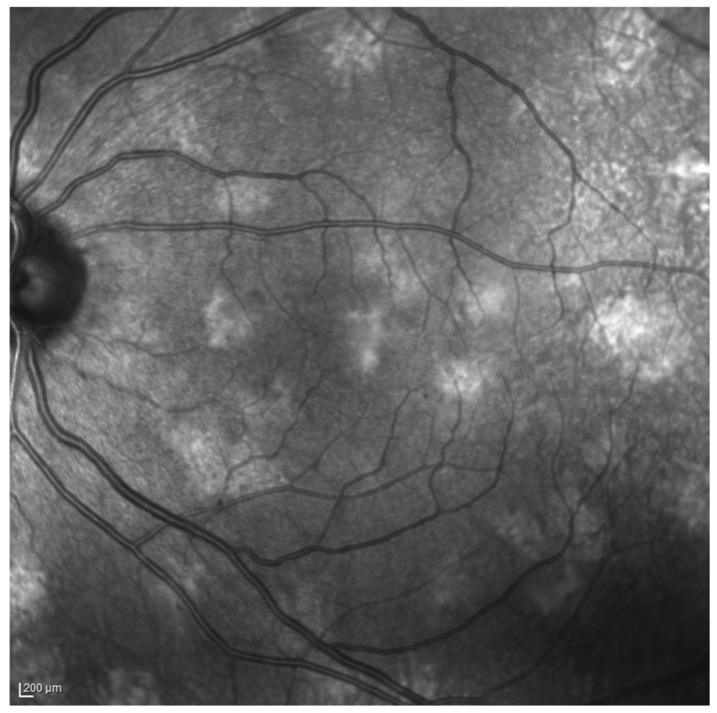
NIR-OCT imaging showing multiple, hyperreflective, patchy CAs in an NF1 patient. Adapted from Moramarco et al. [31].

Subsequently, the spectral domain OCT (SD-OCT) in near-infrared reflectance (NIR) modality, a non-invasive, sensitive and reproducible exam, enables the superior visibility of CAs defined as multiple, bright, patchy nodules (Figure 1) [14]. The advantage of NIR imaging for recording pathological features under the retinal pigment epithelium is due to better transmission of long-wavelength light through melanin and lipofuscin compared to visible light, thereby showing deeper tissues [15]. Thus, the finding of bright, patchy regions under infrared light and the related absence of such areas under conventional ophthalmoscopy, FAF and FA, clearly indicates the choroidal location of these abnormalities. The hyperreflectivity of the CAs on NIR-OCT is ascribable to hyperactivity of the melanosomes and/or to an increase in the number of melanocytes in the choroid. This leads to a robust absorption and consequent backscattering of near-infrared light due to the abundant melanin content [16]. Correspondingly, there have been reports indicating that CAs tend to primarily manifest within the main vascular arcades [17]. This appears to be related to the greater thickness of the choroid and the higher proportion of melanocytes in this area [17]. Interestingly, two subtypes of CAs have been identified: one (1) rounded, bright, well-defined and easily identifiable, and the other (2) patchy, dull, irregular and poorly defined. On SD-OCT, these appear as hyperreflective and dome-shaped in the former case or hyperreflective and placoid in the latter [18].

In recent years, optical coherence tomography angiography (OCTA) studies reported areas of hyper-flow in the deep choroid corresponding to bright patches of CAs on NIR imaging [19].

In adult patient cohorts, CAs demonstrated higher prevalence (82–100%) in NF1 compared to iris Lisch nodules (72–86%), the latter regarded so far as the most frequent diagnostic ocular sign in NF1 [13,14,20]. Furthermore, the frequency of manifestations of up to 100% of CAs approached café-au-lait spots (98%), this being the most common diagnostic criterion in NF1 [20,21]. In addition, CAs showed a sensitivity of 97.5% among the NIH diagnostic criteria, which is second only to café-au-lait spots (98%) and higher than iris Lisch nodules (86%), and a specificity reaching 100% [22]. Moreover, high proportions (14–37%) of patients with NF1 have CAs in the absence of iris Lisch nodules, while only a few subjects (2.5–16%) have iris Lisch nodules without CAs [14,20,21,23]. Also, CAs show an earlier age of presentation in NF1 compared to iris Lisch nodules with rates of 64–95% and 41–52%, respectively, in the paediatric population [14,20,21,23,24]. Also, a recent study demonstrated that CAs are more frequent than iris Lisch nodules in paediatric NF1 cohorts, regardless of a molecularly confirmed diagnosis [23]. These observations suggest that the presence of CAs on NIR imaging may be of higher diagnostic significance than detection of iris Lisch nodules and support the inclusion of both CAs and iris Lisch nodules in the revised diagnostic criteria for NF1. Also, it was reported that CAs grow in number and surface with age, with a maximum increase reached during puberty [14,20,21,25,26]. In a recent study, Chilibeck et al. observed a progression in the number and size of CAs in 14 out of 40 NF1 paediatric patients evaluated longitudinally, with the earliest age progression at 4 years and the oldest at 16 years of age [27]. Similar results were presented by the group of Touzé et al. in a large paediatric NF1 population, observing maximal CA progression between the age of 8 and 12 years [26]. To avoid bias due to the growing process of the eye, Cosmo et al. assessed CA dimensions in a large cohort of NF1-affected children by normalising them for the optic disc size, demonstrating a significant increase over time [28]. Interestingly, Touzé et al. observed that the natural history of CAs differed from café-au-lait macules and OPGs, these progressing until the age of 10–12 years, while that of CAs rather resembled that of neurofibromas and iris Lisch nodules, which typically increase in number and size during puberty [26,29]. This is not surprising given the same embryological derivation and histopathological features of CAs, neurofibromas and iris Lisch nodules. To support this observation, positive correlations were observed between the presence of CAs and neurofibromas [30] and between the number of CAs and the number of iris Lisch nodules [25].

## 3. The New Diagnostic Criteria for NF1

The high sensitivity and specificity for disease emerging from recent studies led to CAs inclusion to current diagnostic criteria for NF1, following a review by an international consensus of experts [8]. The criteria for the diagnosis of NF1 were established by a National Institutes of Health (NIH) Consensus Conference in 1988 [32] and subsequently reviewed in 1997 [33]. In detail, a minimum of two of the following clinical features were required for NF1 diagnosis: six or more CALMs, two or more neurofibromas of any type or one plexiform neurofibroma, axillary or inguinal freckling, a distinctive osseous lesion, a first degree relative with NF1, optic glioma, and two or more iris Lisch nodules [33]. These diagnostic criteria have been widely used in the clinical routine since their original formulation; however, they show low sensitivity in children due to the progressive nature of NF1-related manifestations. In addition, the identification of genetic disorders with phenotypes overlapping those of NF1 has raised debate on diagnostic classification (e.g., segmental/mosaic NF1, constitutional mismatch repair deficiency (CMMRD) syndrome, Legius syndrome). These limitations made the revision of the diagnostic criteria necessary.

The revised diagnostic criteria for NF1 were recently published and include a recommendation to modify an existing criterion from “first-degree relative with NF1” to “a parent with NF1” while adding two new criteria: the presence of a heterozygous pathogenic variant in the NF1 gene with a variant allele fraction of 50% in apparently normal tissue such as white blood cells and the presence of two or more CAs on NIR imaging. Further, in the revised criteria, the presence of either two CAs or iris Lisch nodules is sufficient for this criterion. Specifically, CAs are not included as an independent criterion but as an alternative to the presence of iris Lisch nodules. Indeed, the presence of isolated ophthalmological signs, even if bilateral, are more likely to reflect mosaic rather than constitutional NF1 [8] (Table 1). Importantly, the presence of CAs allows for the differentiation of NF1 from Legius syndrome, the RASopathy with the greatest overlap with NF1 [8].

### 3.1. New Recommendations for Molecular Diagnosis

The revised diagnostic criteria now include the detection of a pathogenic NF1 gene variant as an independent diagnostic criterion [8]. This inclusion holds particular relevance in cases where there is an atypical clinical presentation or in young patients who have not yet exhibited the characteristic features commonly associated with NF1. Additionally, it is applicable in prenatal testing.

**Figure 2:**
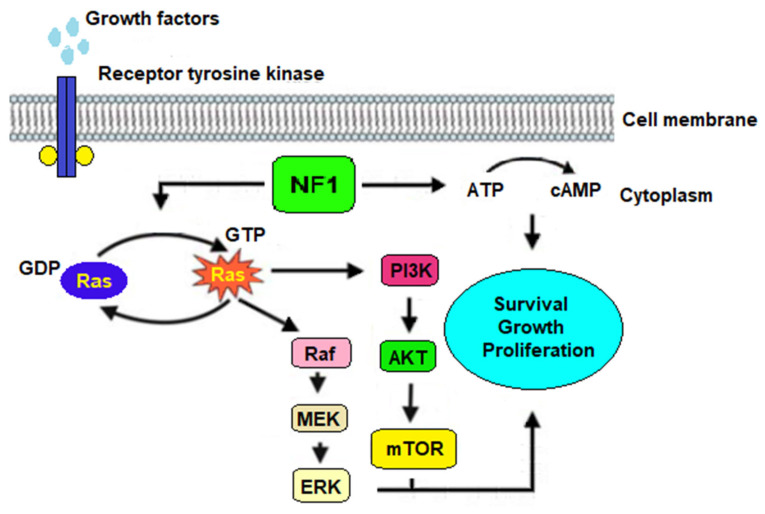
Representative image illustrating the molecular pathogenesis of NF1. NF1 codes for neurofibromin, which is a crucial Ras-GTPase-activating protein. Loss of NF1 expression leads to Ras hyperactivation, which causes the subsequent activation of the Raf/MEK/ERK and the AKT/mTOR pathways. NF1 plays a role also in modulating the ATP-cAMP signalling pathway. The convergence of these pathway activations ultimately leads to aberrant cell growth and uncontrolled proliferation.

The NF1 gene encodes neurofibromin, a tumour suppressor protein that plays a critical role in negative regulation of the RAS pathway by promoting the conversion of active guanosine triphosphate (GTP)-bound RAS to its inactive guanosine diphosphate (GDP)-bound state. Neurofibromin is expressed in neurons, oligodendrocytes and Schwann cells along peripheral nerve trunks. The loss of NF1 function leads to RAS hyperactivation, which in turn triggers a cascade of downstream signalling pathways. This includes the activation of mitogen-activated protein kinase (MAPK), phosphatidylinositol 3-kinase (PI3K), protein kinase B (PKB also known as AKT) and mammalian target of rapamycin (mTOR) kinase. Additionally, NF1 also regulates intracellular cyclic adenosine monophosphate (cAMP) generation. The activation of these pathways has diverse cellular effects, such as cellular proliferation and survival [34] (Figure 2).

Point mutations, such as missense, nonsense and frameshift mutations, in the NF1 gene located on chromosome 17q11.2 are responsible for approximately 90% of NF1 patients. The remaining 5–7% of cases are associated with either a single exon deletion or a complete deletion of the entire NF1 gene [1].

While there have been over 3600 reported pathogenic NF1 variants in NF1, only a limited subset of approximately 30 variants is prevalent enough to be observed in a significant number of affected individuals. Nevertheless, certain genotype–phenotype correlations are starting to emerge [35].

#### 3.1.1. Genotype–Phenotype Correlations

Missense variants affecting p.Met1149 have been associated with a mild phenotype of NF1, characterized by pigmentary features, frequent learning problems and features of NF1-Noonan syndrome [36]. Similar clinical features were observed in p.Arg1038Gly and p.Met992del mutations [37].

In contrast, type 1, 1.4-Mb deletions of the NF1 gene are associated with increased number and earlier appearance of cutaneous and plexiform neurofibromas, a higher risk of developing malignant peripheral nerve sheath tumours, severe cognitive abnormalities, increased cardiovascular anomalies, a recurrent pattern of dysmorphic facial features and other signs of overgrowth including tall stature, large hands and feet [37].

Similarly, missense variants in codons 844 to 848, which encode the cysteine-serine rich domain of the neurofibromin protein, have been observed in individuals with severe phenotypes including plexiform or spinal neurofibromas, optic pathway gliomas, skeletal dysplasia and malignant neoplasms [37,38].

Missense variants affecting p.Arg1809, p.Lys1423 and p.Arg1276 have been associated with a higher frequency of cardiovascular malformations, especially pulmonic stenosis, and the NF1-Noonan phenotype [36,37].

#### 3.1.2. Phenotypic Variants of NF1

Some individuals with NF1 exhibit distinct NF1 phenotypes characterized by unusual combinations of clinical features.

Segmental NF1 is diagnosed in individuals who show typical NF1 features localized to a specific part of the body. In some cases, the involvement of just one part of the body may be a chance occurrence in NF1, especially in young children. In other individuals, segmental NF1 represents results from somatic mosaicism for an NF1 pathogenic variant. Mosaic NF1 usually manifests with less severe symptoms compared to typical NF1 cases involving the same pathogenic variant. Nonetheless, most individuals with mosaicism for an NF1 mutation have been documented to experience a mild, but non-segmental, form of neurofibromatosis. Also, some adults with mosaic NF1 have been reported to be free of clinical features of NF1. Interestingly, adults with mosaic NF1 may have children with typical (non-mosaic) NF1 [39].

The NF1-Noonan syndrome phenotype is observed in around 12% of individuals with NF1. The characteristics of this phenotype may encompass ocular hypertelorism, downslanted palpebral fissures, ptosis, low-set ears, webbed neck, pectus anomaly and pulmonic stenosis [40]. The NF1-Noonan phenotype is genetically heterogeneous. Some individuals have disease-causing variants occurring in both NF1 and PTPN11 (the gene most often involved in Noonan syndrome), while others may have only an NF1 variant. Notably, mutations underlying Noonan syndrome and NF1 are responsible for encoding different proteins that play roles in RAS signalling. The shared involvement of this pathway is thought to be the reason for the phenotypic overlap between the two conditions [41]. Interestingly, Watson syndrome, characterized by pulmonary valvular stenosis, CALMs, short stature and mild intellectual disability, represents an overlapping phenotype with Noonan characteristics, and it is caused by NF1 variants [42].

Among other NF1 phenotypic variants, familial cases of spinal neurofibromatosis, presenting with multiple bilateral spinal nerve root neurofibromas, were reported in the presence of large multiexonic deletion in the NF1 gene but no other clinical features of NF1 [43]. Similarly, multiple spinal ganglioneuromas and multiple lipomas were reported in association with a pathogenic NF1 variant, without other clinical features of NF1 [44,45]. In addition, symptomatic optic gliomas and encephalocraniocutaneous lipomatosis were described in patients with no diagnostic features of NF1 [46,47].

#### 3.1.3. Genetic Testing

A multistep mutation detection protocol that identifies >95% of pathogenic NF1 mutations in individuals fulfilling the NIH diagnostic criteria is available and involves RNA-based techniques including RT-PCR, protein truncation testing (PTT) and/or direct cDNA sequencing complemented with fluorescence in situ hybridization [48].

More recently, multiplex ligation-dependent probe amplification (MLPA) has been incorporated as an initial test to rapidly identify patients with single- or multiexon deletions/duplications, as well as those with a complete gene deletion who do not require complete sequencing of the coding region. Therefore, MLPA has been suggested as a complementary approach to RNA-based techniques to empower the mutation detection technology for NF1 [49].

For prenatal diagnosis, genetic testing (either chorionic villus sampling or amniocentesis) can be offered when the precise mutation of an affected NF1 family member is detected. Alternatively, pre-implantation genetic testing, which involves testing embryos after in vitro fertilization, provides the ability to predict disease severity based on family history [34].

## 4. Hyperpigmented Spots (HSs) in NF1

Recent findings from our group reported that CAs, always visible under infrared light, can reach the level of visibility at fundus examination in limited cases depending on degree of pigmentation and level of inward extension in the deep choroid [30].

**Figure 3:**
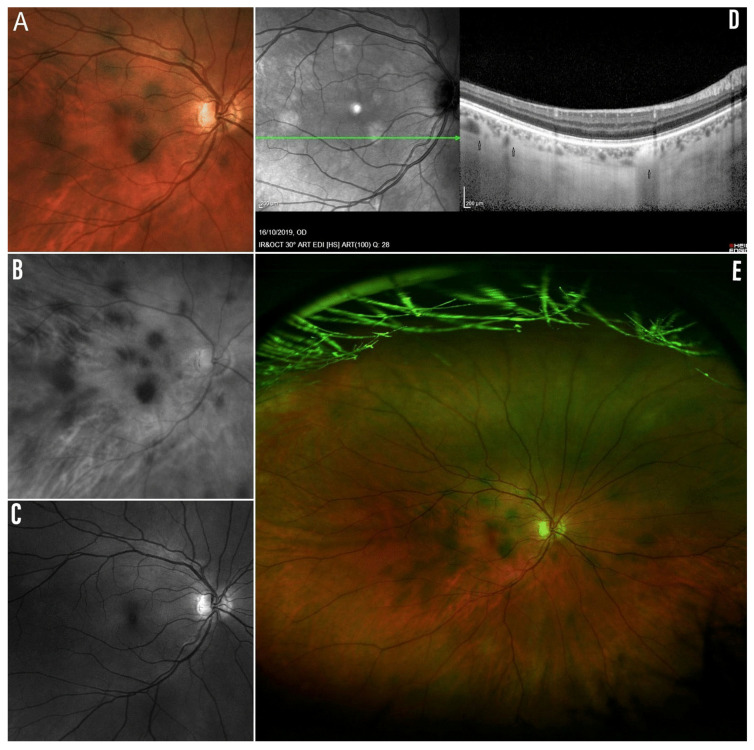
(**A**) HSs on UWF simulated white-light-only image. (**B**) Corresponding UWF red-only laser image showing HSs. (**C**) Corresponding UWF green-only laser image with no spots detection. (**D**) Corresponding NIR-OCT image showing hyperreflective CAs and related inward extension on cross-sectional SD-OCT in EDI modality (black arrow). (**E**) Corresponding UWF pseudocolour composite image (simulated white light, green laser, red laser) showing HSs. Adapted from Moramarco et al. [30].

As evaluated across different wavelengths using ultra-widefield (UWF) scanning laser ophthalmoscopy, NIR-OCT and cross-sectional enhanced depth imaging OCT (EDI-OCT), the most hyperreflective and inwardly extended CAs are visible as hyperpigmented spots (HSs) upon examination of the fundus oculi, thus representing a new ocular sign in NF1 [30]. In support of the above, HSs share a similar appearance and location to CAs, presenting as rounded, hyperpigmented areas of variable size and number with blurred margins and predominant distribution to the posterior pole (Figure 3) [30]. These observations may explain the different frequencies of presentation of CAs and HSs, showing rates of nearly 96% versus 24%, respectively, from our previous results [30].

## 5. Retinal Vascular Abnormalities (RVAs) in NF1

NF1 is associated with a broad spectrum of vascular abnormalities, including stenosis, aneurysms, arteriovenous malformations and hypertension. The frequency of vasculopathy in NF1 is unclear, due to insidious and progressive clinical course. However, findings from large patient cohorts report the prevalence of arterial disease ranging from 0.4% to 10% [50]. Additionally, histological studies reported the presence of cardiovascular disease in nearly 50% of young adults with NF1, placing vasculopathy as the second leading cause of death after cancer in NF1 [51]. The distribution of vascular disease in NF1 is generally irregular and affects multiple vessels [50].

To date, the pathogenesis of NF1 vasculopathy has not yet been clarified. It is well known that loss of neurofibromin leads to the hyperactivation of RAS and downstream effectors, thus making vascular cells more sensitive to different growth factors and thereby contributing to the heterogeneity of disease manifestations observed in NF1 patients [52]. Also, neurofibromin helps maintain the integrity of the endothelial cell layer. Accordingly, it is reported that the loss of neurofibromin in endothelial cells may cause proliferative changes in vascular smooth muscle cells [53]. In addition, neurofibromin deficiency in endothelial cells appears to alter the interaction between endothelial cells and pericytes, perivascular contractile cells, to provide proliferative stimuli for both cell types leading to accelerated angiogenesis in NF1 [54].

**Figure 4:**
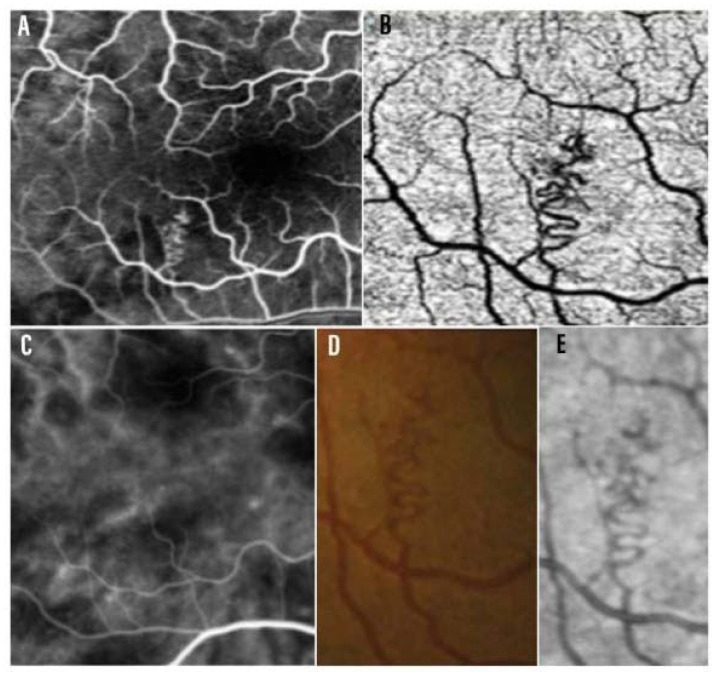
Multimodal Imaging characterization of RVAs: (**A**): arteriovenous phase of fluorescein angiography (FA), (**B**): corresponding RVA captured via OCT Angiography (OCTA) (**C**): Indocyanine Green Angiography (ICGA) (**D**): Color Fundus Photography (**E**): Near-Infrared (NIR) imaging. Scale bar: 200 µm. Adapted from Parrozzani et al. [57].

**Figure 5:**
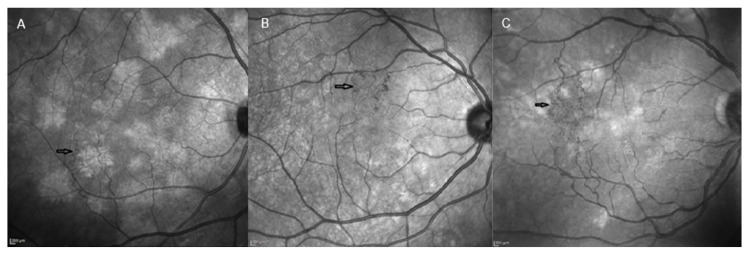
NIR-OCT representative images of simple vascular tortuosity (**A**), corkscrew (**B**) and Moyamoya-like (**C**) RVA patterns in NF1. Scale bar: 200 µm. Adapted from Moramarco et al. [55].

The presence of vascular alterations involving the retinal district in NF1 has been the subject of several studies in recent years. The introduction of NIR-OCT in clinical practice has made it easier to recognize and monitor small vessel alterations compared to indirect ophthalmological examination of the fundus oculi [20]. Retinal vascular abnormalities (RVAs) are also well detectable on FA, showing intraretinal location and absence of leakage, staining or neovascularization [55,56,57,58] (Figure 4). Muci-Mendoza et al. first described the presence of “corkscrew” retinal vessels, a distinctive spectrum of RVAs, consisting of second- or third-order tortuous venules, small tributaries to the superior or inferior retinal veins, with their main location at the posterior pole [56]. The group of Moramarco et al. finally classified RVAs into three main vascular patterns: (1) the simplest and most frequent vascular tortuosity, (2) the “corkscrew” pattern with spiral attitude, and (3) the most complex “Moyamoya-like” pattern (Figure 5) characterised by tortuous vessels that end in a “puff of smoke” arrangement. On the nature of these heterogeneous manifestations, it was speculated that the proliferative attitude involving different vascular wall cells may have led to anatomical modifications and consequent different vascular arrangements. Specifically, differences among RVA models may be related to the different types of involved cells and relative internal or external locations in the vascular wall [55].

The prevalence of RVA varies in studies, between 6.1% and 37.5% [55,56,57,59], while in the paediatric population values are reported ranging from 17.5% to 37.1% [55,58,60]. Specifically, Moramarco et al. reported an overall RVA prevalence of 31.4% in agreement with Abdolrahimzadeh et al. (35%), Muci-Mendoza et al. (37.5%) and Touzé et al. (37.1%) but not with Parrozzani et al. (6.1%) [55,57,59,60]. This variability may possibly be attributed to the different ages of study populations and to the different interobserver agreement for appropriate recognition of RVA patterns. Specifically, the interobserver agreement was mostly low in simple vascular tortuosity, whereas it increased in complex patterns including the “corkscrew”, and “moyamoya-like” vessels [55,56,57,59,60]. In addition, the high diagnostic specificity and the positive predictive value of the corkscrew retinal pattern and moyamoya-like type (100%) made these two signs highly correlated to the diagnosis of NF1 [55].

Muci-Mendoza et al. and Karadimas et al. described RVAs as congenital and stable [56,61]. Nevertheless, recent works by Makino et al. and Touzè et al. reported dynamic changes over time, possibly evolving from simple tortuosity to more complex patterns [56,60,61,62]. In support of the above, a statistically significant correlation was demonstrated between the presence of RVAs and patient age [55]. Interestingly, the group of Touzé et al. observed the evolution of RVAs from simple vascular tortuosity to a corkscrew pattern in 5 cases out of 52 (9%) NF1 children from a retrospective observational study [60]. Based on these findings, the authors investigated the nature of vessel involvement and patterns of progression on near-infrared imaging, reporting tortuous vessels affecting both arteries and veins, while only small second-or third-order venules progressed into a highly tortuous corkscrew pattern, presumably due to their more deformable properties (reported 96% of simple vascular tortuosity, 17% of corkscrew arrangement, no moyamoya-like pattern) [60]. Intriguingly, the high prevalence of evolving RVAs reported by Touzé et al. sensibly differed from the results of Parrozzani et al., which report only 1 patient out of 473 developing de novo RVAs and only 2 patients showing progressive changes during the follow-up from prospective analysis (mean follow-up: 3.7 ± 2.8 years) [63]. This may be explained by the higher average age of the study participants in the study of Parrozzani et al. [63] compared to Touzé et al. [60] (mean age: 17.8 years vs. 8.8 years). These observations suggest that changes in the RVAs may have developmental features in children, with a tendency to stabilise in the more advanced age [64].

Using OCT-A, Parrozzani et al. described the RVAs as isolated tortuous vessels of the superficial vascular plexus associated, in 75% of cases, with a crowded and congested anomalous capillary network of the deep vascular plexus. In addition, anastomotic connections were observed between both arterioles and venules in the superficial capillary complex and the network of congested capillaries in the deep capillary complex. Thus, it was hypothesised that changes in the deep vascular complex may lead to superficial changes in the draining venules due to increased blood pressure and volume, as seen in arterio-venous malformations [57,63]. Based on OCT-A evaluation in 75 patients, the same group proposed a morphological classification of RVAs into three subtypes: (1) macrovascular angiomatosis of the sole superficial vascular complex, (2) macrovascular angiomatosis of the superficial vascular complex combined with microvascular angiomatosis of the deep vascular complex, and (3) combined macrovascular angiomatosis of both superficial vascular complex and deep vascular complex [63]. Consistently, the authors recommended the use of OCT-A over near-infrared imaging for the identification of different types of RVAs [63]. However, we agree with Touzé et al. that OCT-A assessment in the paediatric population could possibly lead to missing RVAs due to technical issues and that, even if RVAs are mostly central, some of them may be located outside of the usual field of OCT-A [60,63]. Additionally, in our view, further large, prospective studies combining NIR-OCT and OCT-A are firmly needed to evaluate the correspondence between the type of macroscopic appearance of RVAs and related type of anatomic location in the retinal plexuses and size of involved vessels, thus allowing for the full morphological characterization of RVAs.

Recent studies also report RVAs as an indicator of ocular and systemic disease complexity [63]. Specifically, a higher prevalence of RVAs has been observed in patients with five or more diagnostic criteria [63]. In accordance, a statistically significant correlation between simple vascular tortuosity and the presence of neurofibromas was demonstrated by our group [55]. Thus, although the presence of RVAs does not appear itself as a negative prognostic factor in visual outcome and clinical implications [55], it may be the sign of higher ocular and systemic disease complexity [63]. In agreement, a possible correlation between the presence of RVAs and severity of NF1 disease should be investigated in future large longitudinal studies.

### 5.1. Additional Vascular Retinal Disorders in NF1

#### 5.1.1. Congenital Abnormalities of the Retinal Vasculature (CARVs)

Among vascular changes involving the retina in NF1, congenital abnormalities of the retinal vasculature (CARVs) have also been reported [65]. In a recent cross-sectional study, the presence of CARVs such as supernumerary optic disc vessels or triple branching of retinal vasculature was demonstrated in 44 out of 48 NF1 patients (91.7%), with 22 patients (45.8%) presenting both findings. The authors speculate that CARV pathogenesis may result from abnormal development of the retinal vasculature in the foetal human eye caused by the loss of NF1 gene function [65]. The sensitivity and specificity of these two CARVs were estimated at 91.7% and 87.5%, respectively. The diagnostic accuracy of the presence of either supernumerary optic disc vessels or triple branching was 0.90. This value was lower than the accuracy of café-au-lait spots (0.98), but higher than that of the other NIH diagnostic criteria, including iris Lisch nodules (0.83) [65]. Interestingly, CARV occurrence was unrelated to the age of patients [65]. The authors assumed that these findings were congenital in origin and non-progressive in nature owing to their pathophysiology [65].

Due to their high sensitivity, specificity and diagnostic accuracy, CARVs were proposed as new ophthalmologic manifestations for an early diagnosis in NF-1 [65]. However, larger, prospective studies are needed to confirm these results.

#### 5.1.2. Ocular Vascular Involvement in Associated Moyamoya Syndrome and NF1

To expand our discussion of NF1-related retinal vasculature involvement, a recent finding is retinal ischemia in patients with associated Moyamoya syndrome and NF1. Moyamoya syndrome is a rare disorder characterised by progressive stenosis and occlusion of small anastomotic vessels in distal branches of cerebral arteries [66]. The development of capillary collateral circulation downstream of the stenotic arterial cerebral vessels produces an image on cerebral angiography that has been described as a “puff of smoke,” or “moyamoya” in Japanese [67].

The ocular vascular anomalies associated with Moyamoya syndrome include anterior ischemic optic neuropathy [68], ocular ischemic syndrome, venous dilation and beading, neovascularization of the retina vessels, vitreous haemorrhage [69], central retinal vein occlusion [70] and central retinal artery occlusion [71].

The occurrence of Moyamoya syndrome associated with NF1 is rare. Fewer than 100 patients with Moyamoya syndrome and NF1 have been reported in the literature, with higher prevalence in children [66,72,73,74]. A possible explanation for this association is that pathogenic genes for Moyamoya syndrome have been identified on chromosome 17, the same pathogenic gene for NF1 [74].

In the ocular district, retinal ischemia secondary to Moyamoya syndrome has been reported, limited to case series, in patients with NF1 [69,75]. Specifically, unilateral ophthalmic artery occlusion was reported in a 12-month-old child diagnosed with associated Moyamoya syndrome and NF1, with evidence of prior unilateral stroke and normal findings on retinal and optic nerve examination at 4 months age [75]. Similarly, signs of ocular ischemia were reported in a 19-month-old boy with NF1. At six years of age, the child developed an acute hemiplegia and was then diagnosed with Moyamoya syndrome [69]. Thus, the severity of ocular and systemic occlusive vascular disease and its progressive nature recommend the close monitoring of children with NF1 to ensure an early diagnosis and treatment of Moyamoya syndrome.

Also, the term moyamoya-like was chosen by Moramarco et al. to identify the morphological aspect of the most complex RVA pattern characterised by tortuous vessels that end in a “puff of smoke” arrangement [55]. In our opinion, given the known association between Moyamoya syndrome and NF1, it would be interesting to evaluate the presence of RVAs, and especially of the moyamoya-like pattern, in patients affected by Moyamoya syndrome and NF1. However, the extreme rarity of these manifestations so far has prevented such an assessment.

### 5.2. The Relationship between CAs and RVAs

It is noteworthy that our research team identified a correlation between HSs and NF1-associated RVAs [31]. Additionally, earlier investigations explored the connection between CAs and concurrent alterations in the retinal microvasculature in individuals with NF1 [59,76].

In recent studies, a topographical correspondence between CAs and overlying corkscrew retinal vessels has been described. This raised a debate on possible pathogenetic hypotheses [59,76]. It was speculated that the development of RVAs could be related to the release of angiogenic factors by the CAs or to functional disorders of vasomotor nerve cells originating in the embryonic neural crest [59,76]. Findings from OCTA studies limited to case series indicate the presence of anomalous retinal vessels situated above CAs in NF1 patients. These abnormalities are characterized by regions of diminished blood flow and decreased vessel density within the choriocapillaris layer [19,77].

A study by Vagge et al., in a case–control design, examined choroidal vascular flows within three patient groups (NF1 patients, healthy controls and NF1 suspects). The authors observed an increased vascular flow area in the superficial choroidal plexus for both NF1 patients and suspected NF1 individuals. However, no significant blood flow differences emerged in the deep choroidal plexus and choriocapillaris. Moreover, an examination of choroidal vascular flow beneath the choriocapillaris was conducted by dividing the deep choroid into distinct levels. In the first choroidal level, there were no notable differences in blood flow among the three groups. In contrast, the second and third levels exhibited reduced flow in patients affected by NF1. These results can probably be explained by an abnormal redistribution of vascular flow caused by the presence of CAs [78].

Recently, an OCTA study of the retinal and choroidal vascular circulation in paediatric patients with NF1 reported significantly reduced perifoveal deep capillary plexus vessel density and choriocapillaris flow (zones of 1 mm, 2 mm and 3 mm around the fovea) area compared with the control group. Moreover, choriocapillaris flow was inversely related to the number of CAs in the 2 mm and 3 mm flow areas, highlighting how choriocapillaris flow rates decrease as the number of choroidal nodules increases. The authors speculate that retinal and choroidal vascular microcirculation are potentially affected by CA compression on the coriocapillaris itself [79].

The results from these papers support the hypothesis that CA compression and the associated blood flow redistribution can possibly have a role in the development of RVAs; however, this needs to be elucidated in further longitudinal studies.

## 6. Neuro-Ophthalmic Manifestations in NF1

### 6.1. Ocular Electrophysiological Findings

Electrophysiological examination is helpful to reveal clinical and subclinical involvement of the central or peripheral nervous system in patients with NF1. Most studies investigating electrophysiological changes in the visual system in NF1 were performed in patients with related optic pathway gliomas (OPGs) [80,81,82].

Specifically, previous studies reported visual evoked potentials (VEPs) as a sensitive method for the screening and monitoring of asymptomatic OPGs in NF1, particularly those involving the chiasm, with reported high specificity (ranging from 55% to 75%) and sensitivity (ranging from 86% to 100%) [80,81,82].

However, as OPGs have a tendency to become symptomatic (only 20–50% of the tumours) before the age of ten, with a median age of onset at 4.4–5.2 years, all routine screening tests, including VEPs, should be applicable to this age group [80]. In this regard, the efficacy of VEPs in the paediatric population is limited due to poor cooperation with the test procedure and a high incidence of false-positive results. Moreover, disease-related cognitive delay and attention difficulties may be additional factors. Either way, VEP test results are crucial for neuroimaging indication, thus resulting in a helpful adjunct to routine clinical ophthalmologic assessment in the screening of OPGs in NF1. The early detection of OPGs then allows for the close monitoring of tumour progression and earlier intervention prior to significant visual loss [80,81,82].

Furthermore, electrophysiological assessment of the neuroretina has been studied in NF1 as an early indicator of damage to the axons that form the optic nerve. Recent findings from experimental studies of murine models of NF1 and OPGs showed optic nerve axonopathy and the apoptosis of retinal ganglion cells occurring in the early stages of OPG development [83]. Consistently, dysfunction of the inner retina was reported in a subgroup of patients with NF1 and OPGs on electroretinogram (ERG) examination compared to the control group [84]. Similarly, Lubiński et al. observed a significant increase in the Arden index in NF1 patients and OPGs under full-field flash ERG and sensory electrooculogram (EOG), while flash ERG examination showed no abnormalities [85,86]. The reported EOG changes were attributed to a dysregulation of the melano-cytogenesis, inducing a disruption in Ca2+ ion flux and an abnormal polarisation of the RPE [85,86]. Interestingly, the group of Touzé et al. explored a possible correlation between RPE dysfunction and the presence of CAs in patients with NF1, showing no statistically significant results [87].

Electrophysiological alterations were also observed in the absence of optic gliomas in NF1 patients. Specifically, VEP abnormalities were reported in NF1 independently of the presence of gliomas of the optic pathways or of the brain. [88]. These findings were ascribed to abnormal visual processing due to dysmyelination of the optic nerve and central nervous system secondary to impaired neurofibromin activity [89]. Specifically, recent evidence shows that the regulatory activity of neurofibromin on the RAS intracellular pathway is exerted also in Schwann cells [89]. Loss of neurofibromin activity in these cells has been shown to dramatically reduce myelination. Therefore, a defect of myelination may explain, at least in part, the observed VEP abnormalities [90]. In accordance, Nebbioso et al. demonstrated a subclinical impairment in the conduction of visual stimuli in patients affected by NF1 and the absence of any condition affecting the optical pathways, as evaluated with VEPs and frequency-doubling technology (FDT) campimetry [90].

In a very recent study, Moramarco et al. reported multifocal-ERG changes in patients without OPGs, with a significant reduction in the P1 wave in all four quadrants compared to healthy subjects. These findings are likely attributable to an alteration in cyclic adenosine monophosphate production resulting in impaired intracellular signal transduction [91].

In the presence of abnormal electrophysiological tests, however, it should be known that sporadic cases of retinal dystrophies have also been reported in association with NF1. Specifically, cone-rod dystrophy has been reported in association with NF1 in two case reports [92,93]. This rare association was suggestive of a cone-rod dystrophy gene situated close to the NF1 gene on chromosome 17 [94]. Also, other retinal dystrophies including adult-onset foveomacular vitelliform dystrophy, retinitis pigmentosa and Usher syndrome have been reported in association with NF1 limited to case reports, with less clear causative relationships [95,96,97].

### 6.2. OCT Evaluation

In addition to electrophysiological studies, OCT evaluation has also been used as a possible diagnostic tool to predict neuro-retinal anomalies in NF1. Very recently, OCT measurement of the peripapillary retinal nerve fibre layer (RNFL) was suggested as a new screening tool for OPGs showing higher diagnostic power compared to VEPs in a large cohort of NF1 patients [82]. Specifically, RNFL thickness analysis had a higher area under the curve (AUC) for discriminating between patients with and without OPGs compared to VEPs (AUC = 0.758; AUC = 0.712, respectively), as well as high specificity (83.3% vs. 63.8%, respectively) and suboptimal sensitivity (65.3% vs. 69.6%, respectively) [82].

Moreover, the evaluation of macular RNFL and ganglion cell layer-internal plexiform layer (GCL-IPL) thickness in NF1 children with OPGs recently demonstrated superiority to peripapillary RNFL evaluation to discriminate between low visual acuity and normal visual acuity, since it is not affected by optic nerve swelling and blood vessel artefacts [98]. In the adult population, a significant reduction in the peripapillary RNFL, macular RNFL and GCL-IPL thickness was found in the absence of optic pathway diseases in NF1 compared to healthy controls, which suggests neuronal and axonal loss [99].

## 7. Conclusions

NF1 represents a challenge for ophthalmologists in terms of early diagnosis, clinical characterization and treatments. In the last few years, considerable advances in multimodal imaging in ophthalmology have led to the description of new ocular manifestations in NF1 including choroidal abnormalities (CAs), hyperpigmented spots (HSs) and retinal vascular abnormalities (RVAs).

Notably, the presence of CAs demonstrates higher diagnostic power compared to the detection of iris Lisch nodules in NF1, especially in the paediatric population. Accordingly, the diagnostic criteria for NF1 have recently been revised to include the presence of two or more CAs on NIR imaging alternatively to the presence of iris Lisch nodules.

Furthermore, the growing knowledge in electrophysiology and OCT, as well as advances in molecular studies, has recently allowed for the identification of subclinical alterations of the visual pathways related to NF1 regardless of the presence of OPGs, with a possible role of neurofibromin in the myelination process.

Therefore, from the numerous studies discussed in this review, there is evidence that research is currently focused on the characterization of novel NF1-related clinical signs in the ocular district, especially in the posterior segment of the eye. This will hopefully allow for an earlier diagnosis and treatment of this disease, with special attention to the paediatric population.

Accordingly, further longitudinal studies are firmly needed to provide additional data on the clinical significance of these new signs involving the ocular system.

Methods of Literature Search

We conducted a search of the literature via PubMed (15 December 2022) using the keywords: “Neurofibromatosis type 1”, “von Recklinghausen’s disease”, “iris Lisch nodules”, “choroidal abnormalities”, “NF1 diagnostic criteria”, “molecular diagnosis”, “genetic testing”, “hyperpigmented spots”, “retinal vascular abnormalities”, “congenital vascular abnormalities”, “Moyamoya syndrome”, “optic pathway gliomas”, “neuro-ophthalmic manifestations”, “Optical coherence tomography”, “OCT Angiography” and “electrophysiology”. No lower time limit was set for the search. Relevant and historical references within the retrieved articles were included. We reviewed English-language articles and non-English-language articles that had translated abstracts. Mendeley software (https://www.mendeley.com/ accessed on 7 August 2023) was used to format the references.

## Figures and Tables

**Table 1:** The revised diagnostic criteria for NF1 [8].

A: The diagnostic criteria for NF1 are met in an individual who does not have a parent diagnosed with NF1 if two or more of the following are present:
Six or more café-au-lait macules over 5 mm in greatest diameter in prepubertal individuals and over 15 mm in greatest diameter in postpubertal individuals Freckling in the axillary or inguinal regions *^1^ Two or more neurofibromas of any type or one plexiform neurofibroma Optic pathway glioma Two or more iris Lisch nodules identified via slit lamp examination or two or more choroidal abnormalities (CAs)—defined as bright, patchy nodules imaged using optical coherence tomography (OCT)/near-infrared reflectance (NIR) imaging A distinctive osseous such as sphenoid dysplasia *^2^, anterolateral bowing of the tibia or pseudarthrosis of a long bone A heterozygous pathogenic NF1 variant with a variant allele fraction of 50% in apparently normal tissue such as white blood cells
B: A child of a parent who meets the diagnostic criteria specified in A merits a diagnosis of NF1 if one or more of the criteria in A are present

*^1^ If only café-au-lait macules and freckling are present, the diagnosis is most likely NF1, but exceptionally, the person might have another diagnosis such as Legius syndrome. At least one of the two pigmentary findings (café-au-lait macules or freckling) should be bilateral. *^2^ Sphenoid wing dysplasia is not a separate criterion in case of an ipsilateral orbital plexiform neurofibroma.
